# The Effect of Solvents on the Crystal Morphology of Isosorbide Mononitrate and Its Molecular Mechanisms

**DOI:** 10.3390/molecules29020367

**Published:** 2024-01-11

**Authors:** Penghui Li, Guimin Zhang, Zongyi Zhou, Ying Sun, Yan Wang, Yu Yang, Xiaolai Zhang

**Affiliations:** 1 School of Chemistry and Chemical Engineering, Shandong University, Jinan 250100, China; 202212239@mail.sdu.edu.cn; 2 National Engineering and Technology Research Center of Chirality Pharmaceutical, Linyi 276006, China; gmzhanglunan@163.com (G.Z.); zky_123789@126.com (Y.Y.); 3 Lunan Pharmaceutical Group Co., Ltd., Linyi 273400, China; lunanzhiyaojituan@126.com (Z.Z.); sunying1982@126.com (Y.S.); 4 Library of Linyi University, Linyi University, Linyi 276000, China; ln8336068@163.com

**Keywords:** 5-ISMN, molecular dynamics simulation, modified attachment energy model

## Abstract

In this work, the modified attachment energy model was used to predict the crystal morphology of isosorbide mononitrate (ISMN) in the dichloromethane (CH_2_Cl_2_) solvent system and dichloromethane-n-hexane (CH_2_Cl_2_-C_6_H_14_) mixed solvent system. The solvent effect can significantly affect the crystal morphology, which can profoundly impact both the drug’s physicochemical properties and the subsequent technological treatment process. In addition, the interactions between solvent molecules and crystal faces were investigated using molecular dynamics simulation, and radial distribution function (RDF) analysis was performed to determine the types of interactions. The structural parameter S was introduced to characterize the roughness of each crystal surface; the change in the CH_2_Cl_2_ diffusion coefficient before and after the addition of C_6_H_14_ was analyzed using mean square displacement (MSD). The calculation results of the modified attachment energy from the two solvent systems revealed that C_6_H_14_ could accelerate crystal growth, while the crystal morphology was not greatly affected, which is of some significance as a guide for the industrial crystallization process.

## 1. Introduction

In the pharmaceutical industry, crystallization is a key step in controlling the crystal habit, which affects the solubility [1], dissolution rate [2], and bioavailability of the drug [3]. At the same time, crystallization is also a key factor affecting postprocessing procedures, such as fluidity [4], stability [5,6], and tableting performance [7,8]. Ren et al. investigated the differences in solubility and dissolution rate of five different crystal habits of ticagrelor (TICA) type II crystals (TICA-A, TICA-B, TICA-C, TICA-D, and TICA-E) in hydrochloric acid solution at pH = 1.2 [9]. Phan et al. prepared two different crystal habits of sorafenib tosylate (Sor-Tos) and investigated their dissolution rates in water and gastric juice pH = 1.2 acid solution [10]. Due to the poor mobility of fine needle-like crystals and the tendency to clog and agglomerate during the formulation process, Pu et al. avoided the formation of needle-like crystals of the glycopeptide vancomycin by controlling the pH and salt concentration [11]. The crystal morphology of ISMN is needle-like, with poor fluidity and low packing density, which brings inconvenience to the subsequent process treatment process [12]. After chemical synthesis of ISMN, the product is purified and refined by solution crystallization [13], and in the process of solution crystallization, different solvents have a large effect on the crystal morphology due to the different sensitivity of crystal faces to solvents [14]. The study of the molecular mechanism of ISMN crystal habit manipulation is important for improving the crystallization process and enhancing product properties.

As a vasodilator, ISMN has broad market prospects because of its pharmacokinetic advantages [15], such as no first-pass effect, high bioavailability, and long duration of action. At present, studies on ISMN mainly focus on pharmacokinetics and synthesis routes [16], but there are few studies on its crystallization and its influence mechanism. Cao et al. investigated the solid–liquid equilibrium behavior of 5-ISMN in different solvent systems and its nucleation behavior in the metastable region, revealing the influence of solvents on the nucleation behavior of 5-ISMN [12]. In this work, the crystal morphology of 5-ISMN in CH_2_Cl_2_ and mixed solvent (CH_2_Cl_2_-C_6_H_14_) was investigated, and the mechanism of the solvent effect on the crystal morphology of 5-ISMN was explained at the molecular level.

With the development of molecular simulation technology, the research method of studying the effect of solvent on crystal morphology by observing crystals cultured from experiments has become a thing of the past. Molecular simulation mainly includes molecular dynamics methods and Monte Carlo methods, which have been widely used in various fields. Song et al. used molecular dynamics simulations for solvent selection to achieve crystal morphology regulation of propionamide [17], and Chen et al. used the Monte Carlo method to predict the adsorption capacity of water molecules on the surface of ammonium dinitramide (ADN) crystals [18]. Additionally, HABIT software can be used for crystal morphology prediction, such as HABIT98 and HABIT95 [19]. HABIT software uses the atom–atom approximation to determine intermolecular interactions in molecular crystals and further calculates the attachment energy. The crystal morphology is modeled by the attachment energy [20]. Roberts et al. used HABIT98 software to study lattice energies and constituent intermolecular interactions for the crystal structures of pharmaceuticals [21]. The program Mercury, developed at the Cambridge Crystallographic Data Centre, has become a powerful platform delivering analysis, design, and prediction functionality alongside visualization for crystal structure [22]. Childs et al. used the Materials module of Mercury CSD to analyze 50 crystal structures containing carbamazepine [23]. In this work, molecular dynamics simulation was used to study the crystal morphology of 5-ISMN.

## 2. Results and Discussion

### 2.1. Intermolecular Interactions within the Crystal Cell

**Figure 1:**
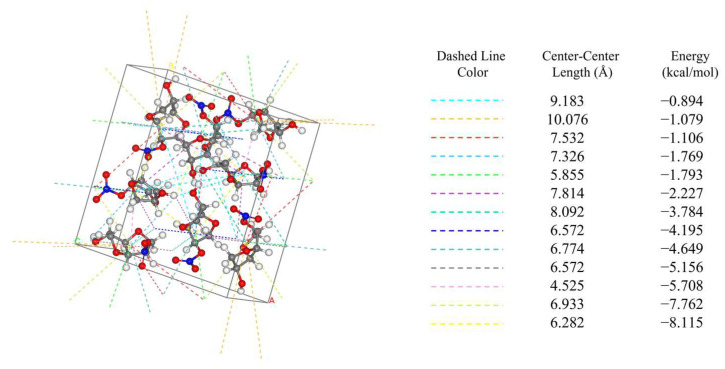
Intermolecular interactions in the unit cell of ISMN.

The attachment energy model (AE model), which is based on the theory of periodic bond chains (PBC) and takes into account the anisotropic properties in the crystal unit [24], is widely used for the prediction of crystal morphology. A detailed discussion of the AE model and the modified attachment energy (MAE) model is provided in Section 3. The AE model determines the relative growth rate by calculating the intermolecular interactions within the crystal cell. The direction and bond energies of intermolecular interactions within the crystal cell are shown in Figure 1.

According to the AE model, the weaker the intermolecular interaction force is, the slower the growth, and the plane corresponding to this force is of greater morphological importance [25].

### 2.2. Crystal Morphology in Vacuum

**Figure 2:**
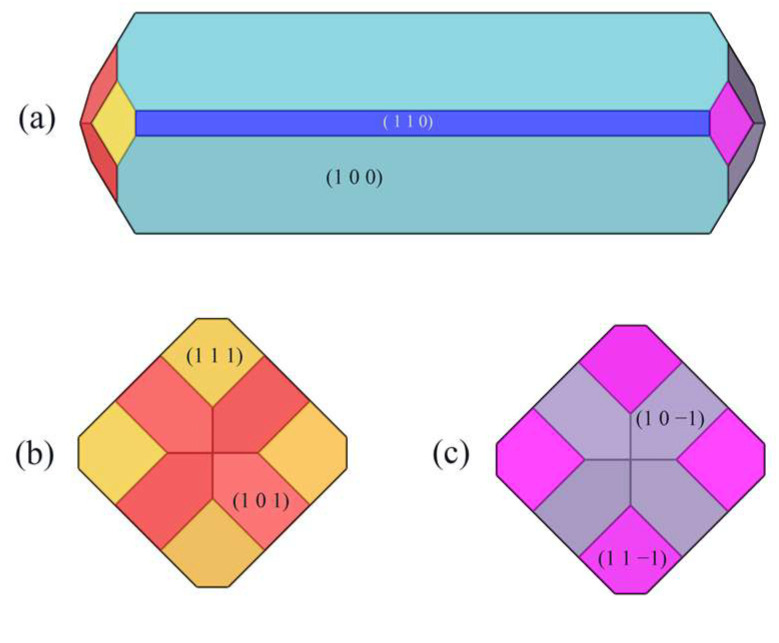
Crystal morphology in vacuum using AE model: (**a**) front view, (**b**) left view, (**c**) right view.

The crystal morphology under vacuum was predicted using the AE model, which had six morphologically important growth surfaces, as shown in Figure 2. The Miller index (h k l) of one face is selected from all of the symmetry images to represent all symmetry-related facets. The symmetry-related facets are represented by the same color.

The interplanar distances of the six morphologically important growth surfaces, the attachment energy under vacuum, and the crystal face areas are shown in Table 1.

The absolute value of the attachment energy (|E_att_| of the (1 0 0) crystal surface is the smallest; the lattice plane spacing (d_hkl_) of the (1 0 0) surface is the largest, which has the greatest morphological importance; and the total crystal face area ratio is up to 72.56%.

### 2.3. Structural Properties of Crystal Faces

The structure of the crystal face closely affects the interaction between the solvent and the crystal face [26]. The rugosity S [27] is introduced to quantitatively characterize the roughness of the six morphologically important growth surfaces, and S is defined as follows:

(1) $$S=\frac{A_{acc}}{A_{hkl}}$$

A_acc_ is the solvent-accessible area and A_hkl_ is the cross-sectional area of the crystal face in the unit cell.

**Figure 3:**
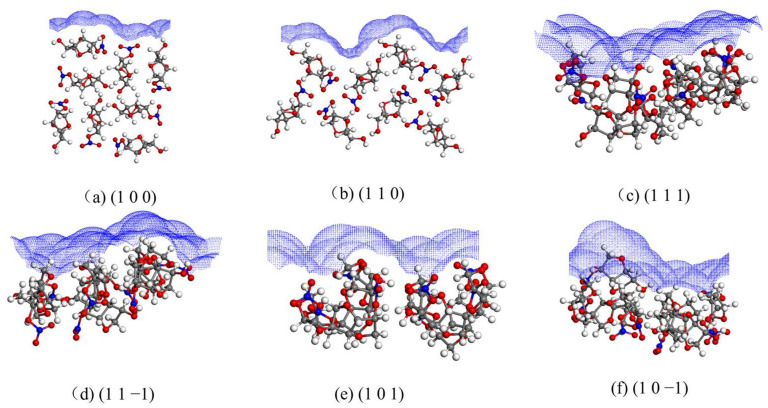
Solvent-accessible area of each crystal face.

The solvent-accessible area of each crystal face is shown in Figure 3.

The rugosity S of each crystal face are shown in Table 2.

The (1 1 0), (1 1 1), and (1 1 −1) crystal faces have similar and large roughness, and the (1 0 0) crystal face has less roughness.

### 2.4. Effect of Solvent CH_2_Cl_2_ on the Morphology of ISMN

#### 2.4.1. Crystal Morphology in the CH_2_Cl_2_ Solvent System

ISMN has high solubility in CH_2_Cl_2_ solvent, which is often used as an extractant to determine ISMN in plasma using gas chromatography [28]. In this work, CH_2_Cl_2_ was chosen as the solvent to study the influence of the solvent on the crystal morphology of ISMN.

Based on the last 100 frames of the conformations of the molecular dynamics simulations, the interaction energies of the solvent layers and crystal face layers of the six crystal face systems were calculated, and the average values were taken as the interaction energy between the solvent layer and the crystal face layer (E_int_) of the corresponding systems. The modified attachment energy term (E′_att_) of the six morphologically important growth surfaces was calculated when the solvent was CH_2_Cl_2_, and the results of the calculations are shown in Table 3.

**Figure 4:**
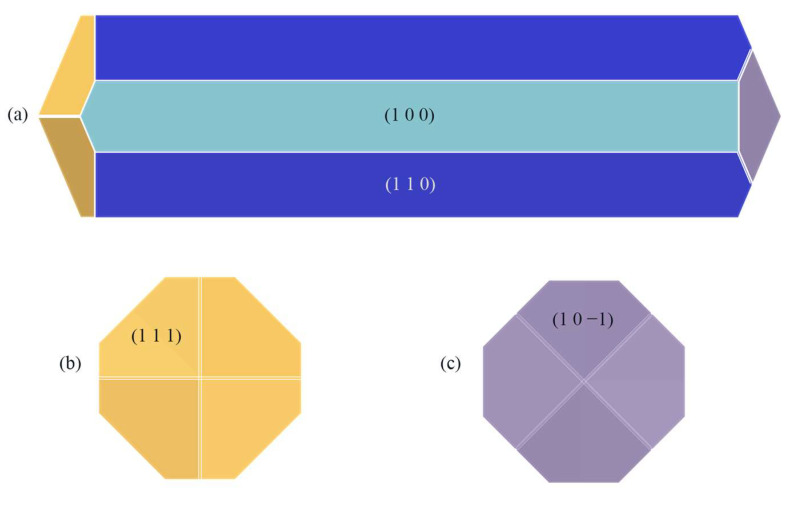
ISMN crystal morphology in the CH_2_Cl_2_ solvent system predicted using the MAE model: (**a**) front view, (**b**) left view, (**c**) right view.

By calculating E’_att_ when the solvent is CH_2_Cl_2_, the crystal morphology was predicted, as shown in Figure 4.

Compared with the crystal morphology under vacuum, the crystal morphology in the CH_2_Cl_2_ solvent system changed somewhat, with the aspect ratio changing from 3.944 in vacuum to 3.886 in the CH_2_Cl_2_ solvent system, and the relative surface area-to-volume ratio changed from 1.343 in vacuum to 1.315 in the CH_2_Cl_2_ solvent system. Meanwhile, the (1 0 1) crystal face and the (1 1 −1) crystal face no longer appeared as morphologically important growth faces in the CH_2_Cl_2_ solvent system.

#### 2.4.2. Diffusion Coefficient

The diffusion coefficient is an important index to characterize the diffusion ability. The diffusion coefficient D can be calculated by Einstein’s diffusion equation, as shown in Equation (2), which can be fitted from the mean square displacement (MSD) [29,30].

(2) $$D=\frac{1}{6}\underset{t\to \infty }{\lim}\frac{d}{dt}\sum_{i=1}^{N}\langle {\left|r_{i}\left(t\right)-r_{i}\left(0\right)\right|}^{2}\rangle$$

**Figure 5:**
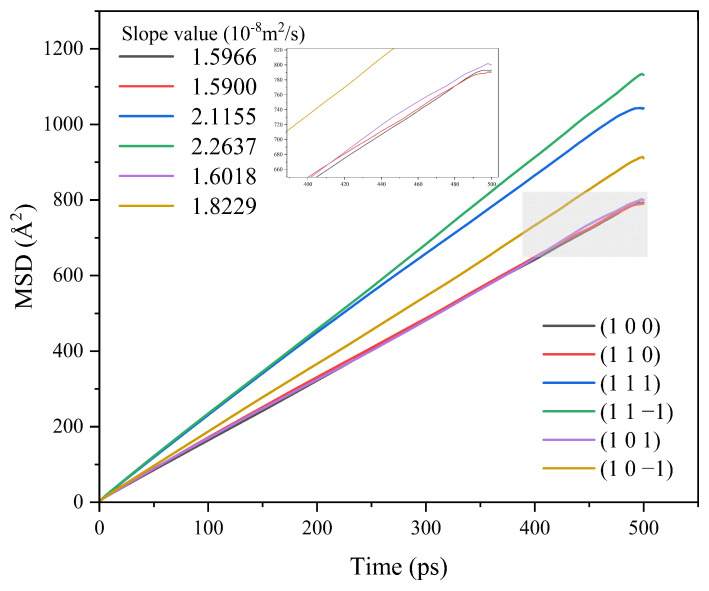
MSD curves of solvent CH_2_Cl_2_ in each crystal face simulation system.

Different crystal face structures affect the diffusion ability of solvent molecules, thus affecting the interaction between the solvent and the crystal face. The MSD analysis of the kinetic simulation trajectories is beneficial to understanding the influence of the diffusion ability of the solvent on the crystal morphology. The MSD curves of solvent CH_2_Cl_2_ in different crystal face systems are shown in Figure 5.

The MSD curves were fitted to obtain the diffusion coefficients of the solvent molecule CH_2_Cl_2_ at different crystal faces, and the diffusion coefficient magnitude relationship was (1 1 −1) > (1 1 1) > (1 0 −1) > (1 0 1) > (1 0 0) > (1 1 0).

#### 2.4.3. Radial Distribution Function Analysis

Due to the different structures of each morphologically important growth face, the exposed functional groups are also different, and the type and strength of the interaction between the solvent and each crystal face become the key factors affecting the crystal morphology. To reveal the essence of the interactions between crystal faces and solvent molecules, RDF [31,32,33] analysis is performed. The RDF is defined as the ratio of the density of the counted atoms within the shell layer at a distance r from the reference atom relative to the average density of the counted atoms in the whole simulation box, and it reflects the type of interaction to some extent.

**Figure 6:**
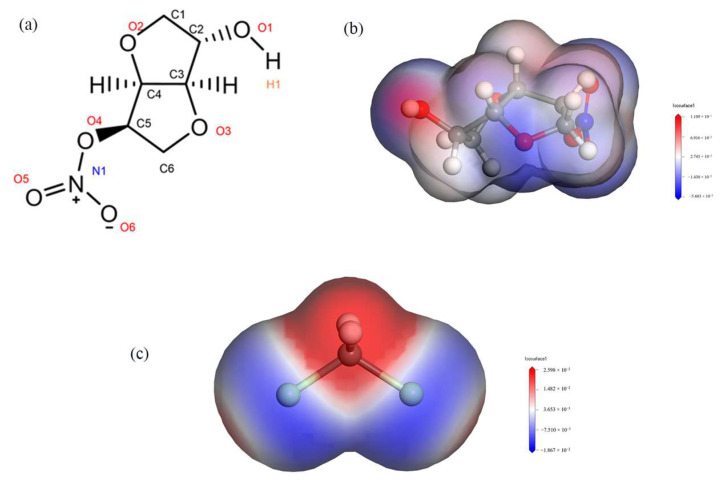
(**a**): Molecular structure of ISMN. (**b**): Electrostatic potential of ISMN. (**c**): Electrostatic potential of CH_2_Cl_2_.

By calculating the electrostatic potentials of ISMN and CH_2_Cl_2_, it was found that the H1 atom in the ISMN molecule has a large positive charge, while the Cl atom in the CH_2_Cl_2_ molecule has a large negative charge, as shown in Figure 6. The RDF was used to analyze the types of interactions between the H1 and Cl atoms mentioned above.

**Figure 7:**
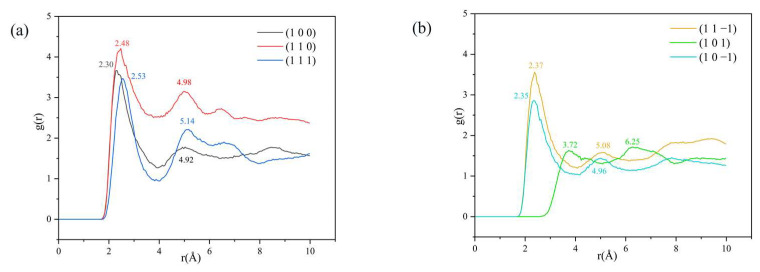
Analysis of the RDF between H1 and Cl atoms: (**a**) (1 0 0), (1 1 0) and (1 1 1) three crystal face systems, (**b**) (1 1 −1), (1 0 1) and (1 0 −1) three crystal face systems.

The reference atom is H1, and the counted atom is Cl, obtaining the radial distribution function between H and Cl atoms, as shown in Figure 7.

From the RDF diagram, it can be seen that a peak exists in the (1 0 0), (1 1 0), (1 1 1), (1 1 −1), and (1 0 −1) crystal face systems in the range of 2.30–2.53 Å, and the magnitude relationship of the peak intensity is (1 1 0) > (1 0 0) > (1 1 −1) > (1 1 1) > (1 0 −1). The hydrogen bond interaction is within 3.1 Å [34,35] and the van der Waals interaction is within 3.1 Å–5 Å [36]. There are hydrogen bonding interactions between H1 and Cl atoms in the above five crystal face systems.

The peak intensity of the RDF in the (1 1 0) crystal face system is significantly larger than that of the other four crystal face systems, indicating the existence of strong hydrogen bonding interactions between H1 and Cl atoms in the (1 1 0) crystal face system; the peak intensity of the (1 0 −1) crystal face system near 2.35 Å is weak, indicating the existence of weak hydrogen bonding interactions. The absence of a peak in the 3.1 Å range for the (1 0 1) crystal face system indicates that there is no hydrogen bonding interaction in this crystal system. The presence of a peak in the 3.1 Å–5 Å range for the (1 0 0), (1 1 0), (1 0 1), and (1 0 −1) crystal face systems suggests that there are also van der Waals interactions between the H1 and Cl atoms in the above crystal face systems.

#### 2.4.4. Analysis of Hydrogen Bonding Interactions

**Figure 8:**
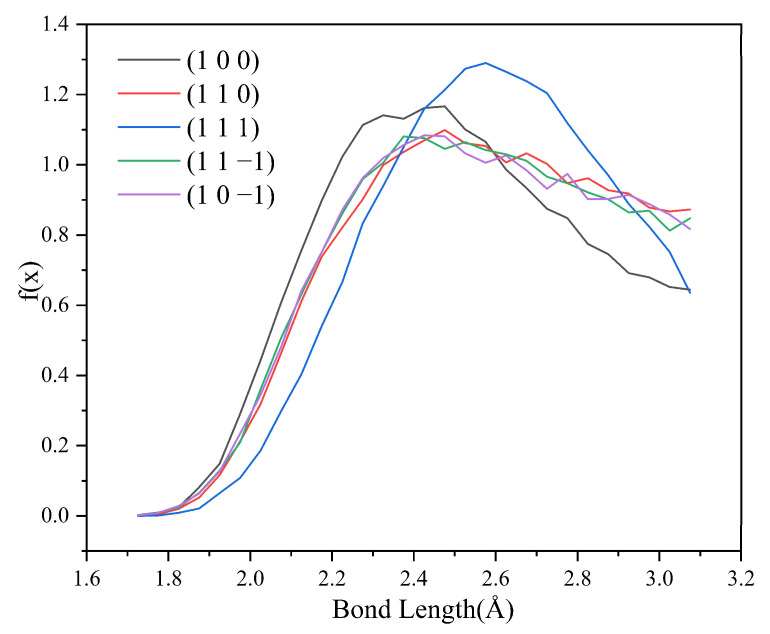
Probability density distributions of hydrogen bond lengths.

Since no hydrogen bonding interaction is formed between the (1 0 1) crystal face and the solvent molecules, to reveal the effect of hydrogen bonding interaction on the crystal morphology, hydrogen bonding statistics were performed for the last 300 ps of the molecular dynamics simulation trajectories for the remaining five crystal face systems. Probability density distributions of the bond lengths and bond angles of the hydrogen bonds formed between H1 and Cl atoms are obtained, as shown in Figure 8.

From the probability density distribution of hydrogen bond lengths, it can be seen that the probability of hydrogen bond lengths less than 2.42 Å in the (1 0 0) crystal face is higher than that in the other four crystal faces, and the probability that the bond lengths of the hydrogen bond formed on the (1 1 1) crystal face lie in the range of 2.42–3.10 Å is greater than that on the other four crystal faces. The probability density distributions of hydrogen bond lengths are similar for the (1 1 0), (1 1 −1), and (1 0 −1) crystal face systems.

From the probability density distributions of the hydrogen bond angles formed in the five crystal face systems, it can be seen that there is little difference in the probability density distributions of hydrogen bond angles among the three crystal faces (1 1 0), (1 1 −1), and (1 0 −1). Among the five crystal face systems, the probability density distributions of hydrogen bond lengths and angles on the (1 1 1) crystal face both have the highest peaks, indicating that the distribution range of hydrogen bond lengths and angles on the (1 1 1) crystal face is relatively concentrated, and the bond lengths and angles of hydrogen bonds are more likely to occur at approximately 2.57 Å and 150°, respectively.

The angle between D-H⋯A (D is the donor, A is the acceptor) is straight or close to 180°, and the shorter the distance between H⋯A, the more stable the individual hydrogen bonding interaction formed [37]. The hydrogen bonding statistics are shown in Table 4: it can be seen that the individual hydrogen bonding interaction formed by the (1 0 0) crystal face is more stable.

#### 2.4.5. Effect of Hydrogen Bonds on Changes in Crystal Morphology

The change in crystal morphology was analyzed from the hydrogen bond perspective by comparing the crystal morphology under vacuum with that in the CH_2_Cl_2_ solvent system. In the competitive growth process of the (1 1 1) and (1 0 1) crystal faces, the interaction between the solvent CH_2_Cl_2_ molecules and the (1 1 1) crystal face is strong because the (1 1 1) crystal face can form hydrogen bond interactions with the solvent CH_2_Cl_2_ molecules, while the (1 0 1) crystal face does not form hydrogen bond interactions with the solvent CH_2_Cl_2_ molecules. The growth of solute molecules in the (1 1 1) crystal plane is hindered, so the (1 1 1) crystal face grows slowly and shows greater morphological importance in the solvent environment.

The area percentage of the (1 1 0) crystal face varies greatly in the two conditions, with an area percentage of 12.85% in vacuum and 48.93% in the CH_2_Cl_2_ solvent system. In the competitive growth process of the lateral crystal faces of (1 0 0) and (1 1 0), the single hydrogen bond formed by the (1 0 0) crystal face is more stable, but the (1 1 0) crystal face has more H1 atoms involved in hydrogen bond formation. Considering that the MS 2018 software predicts the crystal morphology using the MAE of each crystal face in the unit cell, by counting N_HB-hkl_, it is found that the average number of hydrogen bonds contained in the (1 1 0) crystal face in the unit cell is much greater than that in the (1 0 0) crystal face. As a result, the interaction between the CH_2_Cl_2_ solvent molecules and the crystal face on the (1 1 0) crystal face is stronger than that on the (1 0 0) crystal face in general, and the growth of the (1 1 0) crystal face is slower, reflecting greater morphological importance.

**Figure 9:**
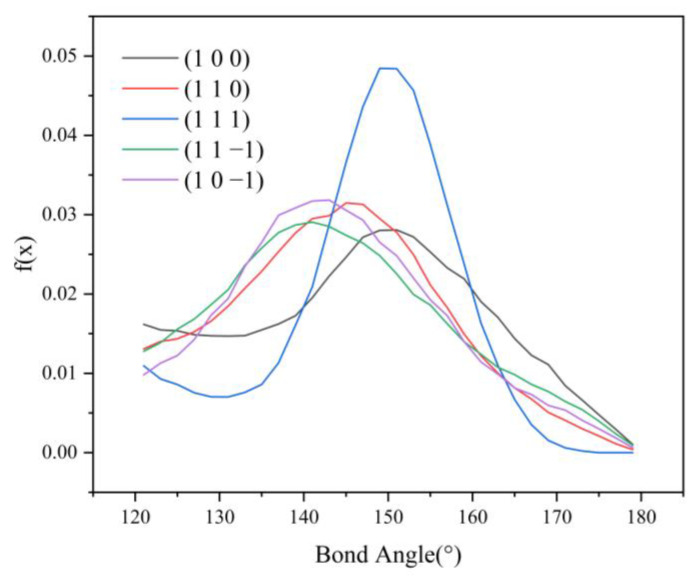
Probability density distribution of hydrogen bond angles.

HB length, HB angle, S_1_%, and S_2_% of the hydrogen bonds formed between the (1 1 −1) and (1 0 −1) crystal faces and the solvent molecules do not differ much. However, the bond angle probability density distribution functions of the two crystal faces show a large difference in the range of 120–150°, with the probability of the bond angle in the range of 120–133° being greater for the (1 1 −1) crystal face (shown by the green line in Figure 9) than for the (1 0 −1) crystal face (shown by the violet line in Figure 9). The probability of the bond angle of the hydrogen bonds formed on the (1 0 −1) crystal face is greater than that of the (1 1 −1) crystal face in the range of 133–150°. Under the same conditions, the larger the bond angle is, the more stable the hydrogen bond formed, so the interaction between the (1 0 −1) crystal face and the solvent molecules CH_2_Cl_2_ is stronger and shows greater morphological importance.

### 2.5. Prediction of Crystal Morphology in the CH_2_Cl_2_-C_6_H_14_ Solvent System and Analysis of Results

#### 2.5.1. Crystal Morphology in the CH_2_Cl_2_-C_6_H_14_ Solvent System

Adding n-hexane (C_6_H_14_) to the solution can accelerate the volatilization of the solution and realize volatilization crystallization. Adding C_6_H_14_ to the model solvent layer, the simulation results are discussed.

The E’_att_ of the six morphologically important growth surfaces in the CH_2_Cl_2_-C_6_H_14_ solvent system was calculated using the same method as in the CH_2_Cl_2_ solvent system, and the results are shown in Table 5.

Compared with the CH_2_Cl_2_ solvent system, the absolute value of E’_att_ increased in all six crystal face systems in the CH_2_Cl_2_-C_6_H_14_ solvent system, indicating that after the addition of C_6_H_14_, the interaction between the solvent layer and the crystal face layer is weakened, the interaction between the solute and the crystal face is enhanced, and the growth rate of the crystal increases.

**Figure 10:**
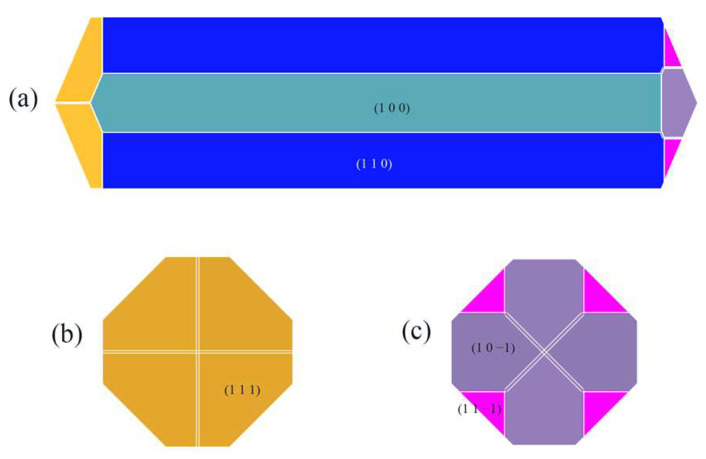
ISMN crystal morphology in the CH_2_Cl_2_-C_6_H_14_ solvent system predicted using the MAE model: (**a**) front view, (**b**) left view, (**c**) right view.

The crystal morphology in the CH_2_Cl_2_-C_6_H_14_ solvent system was obtained by E’_att_ and is shown in Figure 10.

In contrast to the crystal morphology in the CH_2_Cl_2_ solvent system, the (1 1 −1) crystal face reappears as a morphologically important growth surface in the CH_2_Cl_2_-C_6_H_14_ solvent system.

#### 2.5.2. Diffusion Coefficient of CH_2_Cl_2_ in the CH_2_Cl_2_-C_6_H_14_ Solvent System

**Figure 11:**
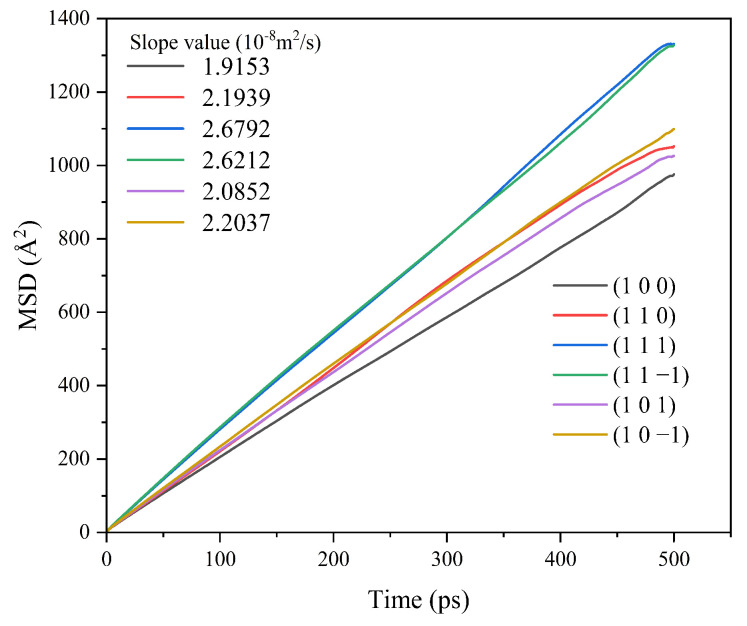
MSD curve of CH_2_Cl_2_ in the CH_2_Cl_2_-C_6_H_14_ solvent system.

The MSD of CH_2_Cl_2_ in the CH_2_Cl_2_-C_6_H_14_ solvent system was analyzed, and the results are shown in Figure 11.

The MSD curves were fitted to obtain the diffusion coefficients of the CH_2_Cl_2_ solvent molecules in the CH_2_Cl_2_-C_6_H_14_ solvent system, and the results are shown in Table 6.

The diffusion coefficients of CH_2_Cl_2_ at all six crystal face systems were increased compared with those before the addition of the volatile agent C_6_H_14_, indicating that the addition of C_6_H_14_ can accelerate the diffusion of CH_2_Cl_2_.

#### 2.5.3. Analysis of Hydrogen Bond Interactions in the CH_2_Cl_2_-C_6_H_14_ Solvent System

**Figure 12:**
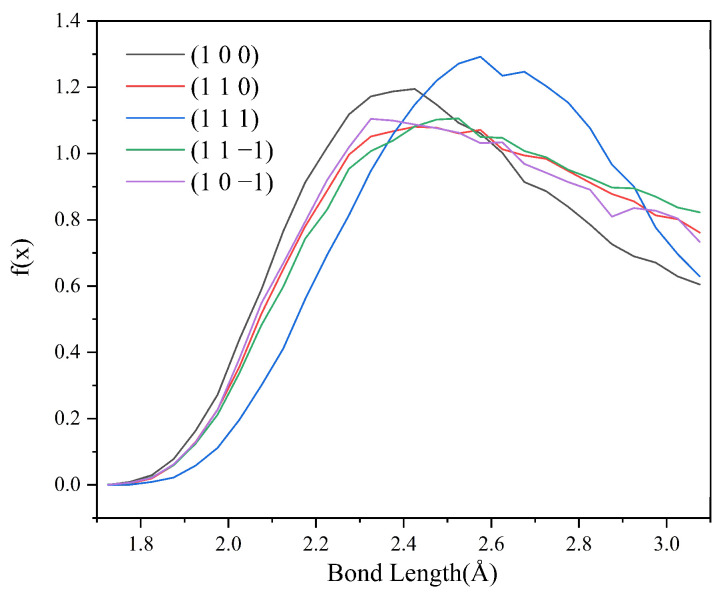
Probability density distributions of HB lengths in the CH_2_Cl_2_-C_6_H_14_ solvent system.

**Figure 13:**
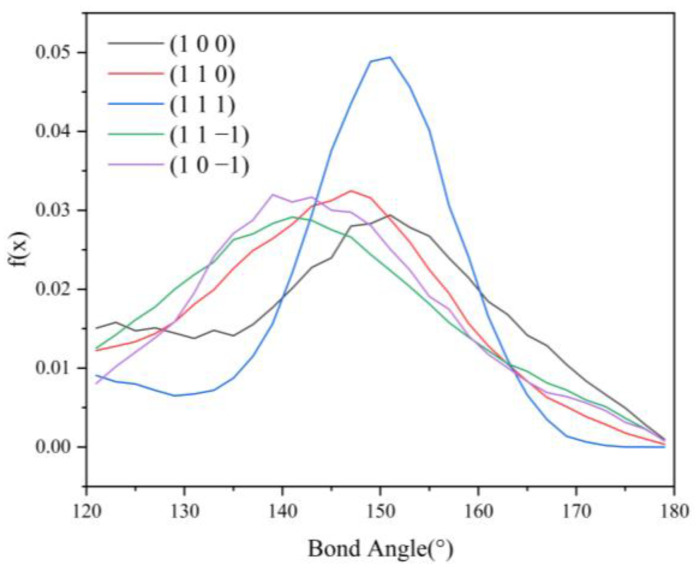
Probability density distributions of HB angles in the CH_2_Cl_2_-C_6_H_14_ solvent system.

Hydrogen bonding statistics were performed for the last 300 ps of the kinetic simulation trajectories of the five crystal face systems (1 0 0), (1 1 0), (1 1 1), (1 1 −1), and (1 0 −1), and the probability density distributions of the bond lengths and angles of the hydrogen bonds formed between the H1 and Cl atoms were obtained, as shown in Figure 12 and Figure 13.

Compared with the CH_2_Cl_2_ solvent system, the probability density distribution characteristics of the hydrogen bond lengths and angles did not change much after the addition of C_6_H_14_, indicating that the addition of the volatile agent C_6_H_14_ did not greatly affect the stability of the hydrogen bonds too much. The hydrogen bonding statistics are shown in Table 7.

From the statistical results, it can be seen that the HB length, HB angle, S1%, and S2% do not change much compared with those before the addition of C_6_H_14_. Except for the (1 1 1) crystal face, the N_HB-unit area_, and N_HB-hkl_ in the other four crystal face systems change considerably, from which it can be hypothesized that the reduction in the number of hydrogen bonds after the addition of the volatile agent C_6_H_14_ leads to the weakening of the interaction between the solvent layer and the crystal face layer. Thus, the absolute value of the E_s_ term in the modified attachment energy model decreases, and the absolute value of E’_att_ increases.

#### 2.5.4. Relative Concentration Distributions of CH_2_Cl_2_ and C_6_H_14_

**Figure 14:**
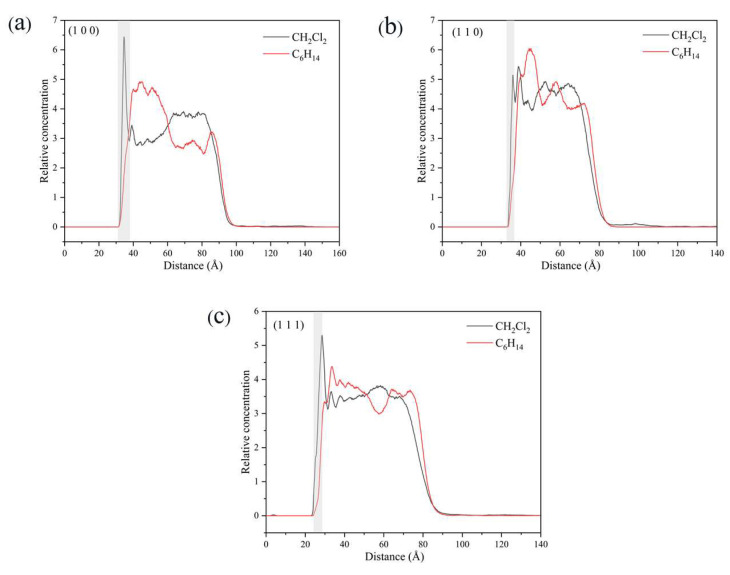
Relative concentration distributions of CH_2_Cl_2_ and C_6_H_14_ in the *z*-axis direction for the three crystal face simulation systems (1 0 0), (1 1 0), and (1 1 1): (**a**) (1 0 0) crystal face system, (**b**) (1 1 0) crystal face system, (**c**) (1 1 1) crystal face system. (The light gray area in the figure shows the relative concentration distribution in the vicinity of the crystal face).

In the vicinity of the crystal face, the interaction between solvent molecules and the crystal face largely determines the crystal morphology, and the relative concentration distributions of CH_2_Cl_2_ and C_6_H_14_ were calculated in the direction perpendicular to the crystal face. By analyzing the last 200 ps of the kinetic simulation trajectory, the relative concentration distributions were obtained. The relative concentration of CH_2_Cl_2_ is high and that of C_6_H_14_ was low near the crystal face in the three crystal face simulation systems (1 0 0), (1 1 0), and (1 1 −1), as shown in Figure 14.

**Figure 15:**
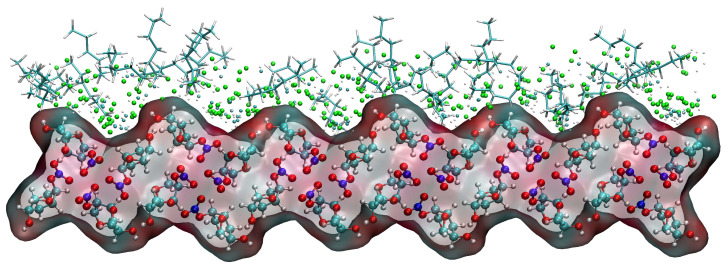
Concentration distribution of CH_2_Cl_2_ and C_6_H_14_ near the (1 1 0) crystal face. (The lower layer is the ISMN. In the upper layer, C_6_H_14_ is represented by a stick model, the Cl atom in CH_2_Cl_2_ by a green sphere, the C atom in CH_2_Cl_2_ by a cyan sphere, and the H atom in CH_2_Cl_2_ by a white sphere.)

Taking the (1 1 0) crystal face as an example to explore the reasons for the higher concentration of CH_2_Cl_2_ solvent molecules near the crystal face, the microstructure near the crystal face during the kinetic simulation is analyzed as in Figure 15. The relative concentration distribution in the figure is at the contact area between the solvent layer and the crystal plane. Part of the solvent molecules above and part of the crystal molecules below outside the contact region have been hidden. The reason for the higher concentration of CH_2_Cl_2_ near the crystal face is mainly due to the existence of small depression regions at the crystal face, and the CH_2_Cl_2_ molecule, because of its smaller molecular structure, is embedded in the depression regions and interacts more strongly with ISMN molecules at the crystal face. The larger molecular structure of C_6_H_14_ has a larger spatial site resistance to enter the depression regions, so its concentration is lower near the crystal face.

The density distributions near the (1 0 0) and (1 1 1) crystal faces are similar to those near the (1 1 0) crystal face, with both higher CH_2_Cl_2_ concentrations and lower C_6_H_14_ concentrations near the crystal face. The molecular structures of CH_2_Cl_2_ and C_6_H_14_ and the spatial site barrier effect at the crystal face combine to contribute to the phenomenon.

**Figure 16:**
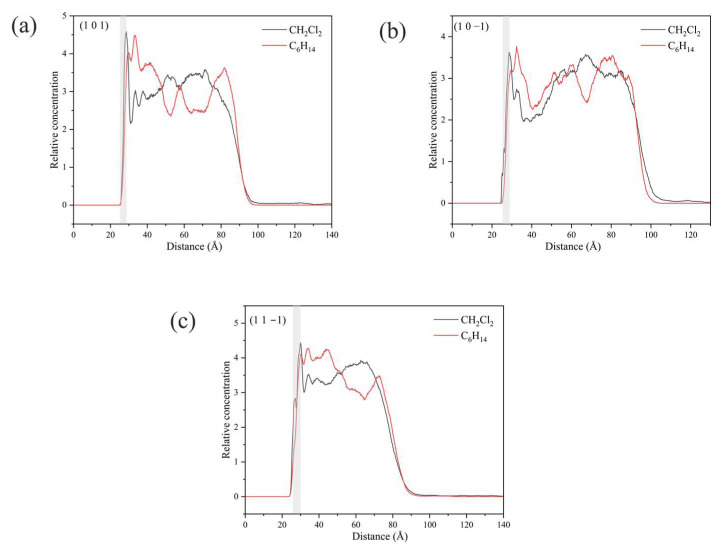
Relative concentration distributions of CH_2_Cl_2_ and C_6_H_14_ in the *z*-axis direction for the three crystal face simulation systems (1 0 1), (1 0 −1), and (1 1 −1): (**a**) (1 0 1) crystal face system, (**b**) (1 0 −1) crystal face system, (**c**) (1 1 −1) crystal face system. (The light gray area in the figure shows the relative concentration distribution in the vicinity of the crystal face.)

Unlike the three crystal faces mentioned above, the relative concentrations of CH_2_Cl_2_ and C_6_H_14_ in the simulated systems of (1 0 1), (1 0 −1), and (1 1 −1) are both high in the vicinity of the crystal faces, as shown in Figure 16.

The high concentration of CH_2_Cl_2_ and C_6_H_14_ at the (1 0 1) crystal face is mainly because CH_2_Cl_2_ is unable to form hydrogen bonding interactions with the crystal surface in this crystal face simulation system, and the interaction between CH_2_Cl_2_ and the crystal face is weaker, resulting in a high concentration of C_6_H_14_ in this region as well.

**Figure 17:**
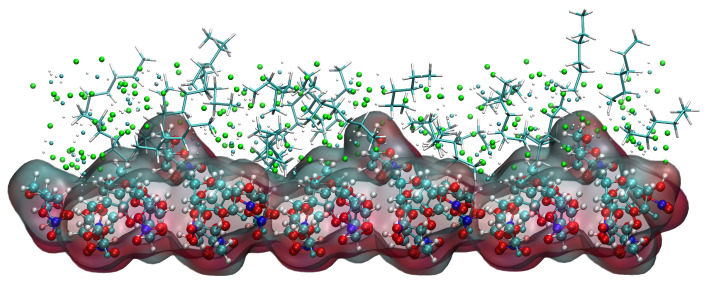
Concentration distribution of CH_2_Cl_2_ and C_6_H_14_ near the (1 0 −1) crystal face. (The lower layer is the ISMN. In the upper layer, C_6_H_14_ is represented by a stick model, the Cl atom in CH_2_Cl_2_ by a green sphere, the C atom in CH_2_Cl_2_ by a cyan sphere, and the H atom in CH_2_Cl_2_ by a white sphere.)

The analysis of the kinetic trajectory of the (1 0 −1) crystal face system reveals that due to the larger space in the depression regions at the (1 0 −1) crystal face, the site resistance of the C_6_H_14_ molecules to enter the region is smaller, so the relative concentration of CH_2_Cl_2_ and C_6_H_14_ peaks in the vicinity of the crystal face. The microstructure near the (1 0 −1) crystal face is shown in Figure 17.

The reason for the high concentration of both CH_2_Cl_2_ and C_6_H_14_ at the (1 1 −1) crystal face is that the depression regions at the crystal face are so small that CH_2_Cl_2_ molecules cannot enter the region either, failing to reflect its structural advantages.

#### 2.5.5. Analysis of Changes in the Crystal Morphology of ISMN in the CH_2_Cl_2_-C_6_H_14_ Solvent System

CH_2_Cl_2_ solvent molecules can form hydrogen bond interactions with ISMN molecules on the (1 0 −1) crystal face, while C_6_H_14_ molecules do not form hydrogen bond interactions with ISMN molecules on the (1 0 −1) crystal face. The high concentration of C_6_H_14_ near the (1 0 −1) crystal face weakens the interaction between the solvent layer and the crystal face layer, and the (1 0 −1) crystal face growth is faster. Therefore, when growing along with the (1 1 −1) crystal face, the (1 1 −1) crystal face has the opportunity to reappear as a morphologically important growth face.

## 3. Simulation

### 3.1. Calculation Methodology

There are three popular methods for studying crystal morphology, namely, the BDFH method, the growth morphology method, and the equilibrium morphology method [17]. The BDFH method [38,39] was first proposed by Bravais, verified by Friedel’s observations, and improved by Donnay and Harker. The theory states that the normal growth rate of a crystal surface is inversely proportional to the lattice plane spacing (d_hkl_). The growth morphology method determines the relative growth rate based on the magnitude of the intermolecular interactions within the crystal and is also referred to as the attachment energy model (AE model) [40,41]. The equilibrium morphology method works by calculating the minimum surface free energy for a given volume and temperature. Wulff plots are combined to visualize the morphology of crystals in equilibrium with their surroundings [42]. Among the above methods, the AE model is widely used for the prediction of crystal habits of energy-containing materials [43] and drug molecules [44] due to its simple computational steps and relatively reliable accuracy, and the AE model is used in this work to predict the crystal morphology.

In the AE model, the relative growth rate (R_hkl_) of a crystal face in vacuum is proportional to the absolute value of the attachment energy (|E_att_|) of the corresponding lattice plane [45,46].

R_hkl_∝│E_att_│ (3)

The crystal plane with the most negative E_att_ will have the fastest growth rate and at the same time the least morphological importance [24,31]. E_att_ is calculated as follows:

E_att_ = E_latt_ − E_slice_ (4)

where E_att_ is the energy released when a wafer of thickness d_hkl_ is attached to the surface of the growing crystal, E_latt_ is the lattice energy of the crystal, and E_slice_ is the energy possessed by a wafer of thickness d_hkl_.

In solution, due to the interaction between the solvent molecules and the crystal surface, the growth of the crystal surface requires the exclusion of the solvent–crystal surface interaction, and the AE model needs to be corrected. In solution, the modified attachment energy (MAE) is calculated as follows:

E′_att_ = E_att_ − E_s_ (5)

E′_att_ is the modified attachment energy term, and E_s_ is the solvent adsorption effect on the attachment energy term, which can be obtained from the interaction energy between the solvent layer and the crystal face layer (E_int_). E_int_ is expressed as follows:

E_int_ = E_tot_ − (E_cry_ + E_sol_) (6)

where E_tot_ is the total energy of the solvent layer and crystal face system, E_cry_ is the potential energy of the crystal face layer alone without the solvent layer, and E_sol_ is the potential energy of the solvent layer alone without the crystal face layer. The unit of measure for all three is kcal/mol.

The unit of measure of the attachment energy E_att_ in Materials Studio (MS) software is kcal/mol/unit cell. To ensure the consistency of E_s_ and E_att_, it is necessary to convert E_int_ by introducing the following conversion factors [47]:

(7) $$E_{s}=\frac{Z_{cry}}{Z_{hkl}}\times \frac{A_{hkl}}{A_{box}}E_{int}$$

E_int_ is the interaction energy between the solvent layer and the crystal face in the simulation box in kcal/mol; A_hkl_ is the cross-sectional area of the crystal face cut out from the unit cell in Å^2^; A_box_ is the cross-sectional area of the simulation box in Å^2^; A_hkl_/A_box_ is the reciprocal of the number of unit crystal faces contained in the simulation box, e.g., to construct a 2 × 3 simulation interface, then A_hkl_/A_box_ = 1/6; Z_cry_ is the number of molecules contained in the unit cell; Z_hkl_ is the number of molecules contained in the crystal face cut from the unit cell.

Through the above conversion, E_s_ and E_att_ have the same dimension kcal/mol/unit cell.

### 3.2. Simulation Methods and Details

#### 3.2.1. Optimization of Lattice Parameters

The original cell structure of the ISMN used in the simulation was obtained by testing and analyzing the product manufactured by Lunan Pharmaceutical Group Corporation, which belongs to the P4_3_ space group, and its lattice parameters are shown in Table 8. The difference is not significant compared with the cell structure obtained from the Cambridge Structure Database [48]. The COMPASS force field was used to optimize the geometry of the original cell, and the degree of variation of the optimized lattice parameters was within the acceptable range. The COMPASS force field was used in this work for the following reasons: Validation studies representing 28 molecular classes show that COMPASS force field enables accurate and simultaneous prediction of structural, conformational, vibrational, and thermophysical properties for a broad range of molecules in isolation and in condensed phases [49]. These 28 molecular classes include C_6_H_14_ and CH_2_Cl_2_, the two solvent molecules in this simulation. The molecular dynamics simulation of organic drug crystals by the COMPASS force has also been effectively verified, for example, the prediction of sulfamerazine crystal morphology [27] and the crystallization of mefenamic acid using N, N-dimethyl formamide (DMF) as a solvent [50]. Cao et al. analyzed the intermolecular interactions of 5-ISMN and four organic solvents using molecular simulations, and the force field used for the molecular simulations was the COMPASS force field [12]. So, we believe that the COMPASS force field is applicable to the simulation system in this work.

#### 3.2.2. Construction of the Simulation System

**Figure 18:**
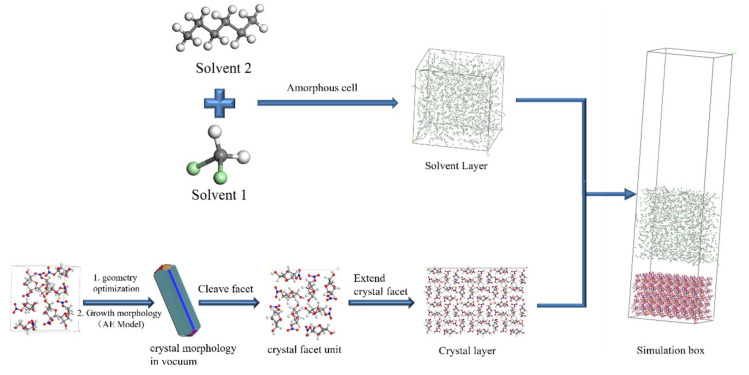
Schematic diagram of the construction of the simulation system.

The AE model was used to predict the crystal morphology of the geometry-optimized crystal cell under vacuum conditions. The morphologically important growth faces are cut out and extended. The simulation box consists of the crystal surface layer and the solvent layer, where the solvent layer is composed of solvent molecules. The construction process of the simulation system is shown in Figure 18.

The size of the simulation box affects the results of molecular dynamics simulations [51,52]. Lan et al. [25] investigated the effect of the size of the interface model of the ɛ-hexanitrohexaazaisowurtzitane (HNIW) binary system on the results of the modified attachment energy calculations. The results showed that the length and width of the simulation box should be not less than twice the truncation radius (a ≥ 2d_c_) and the thickness of the crystal face layer should not be less than the truncation radius (T_c_ ≥ d_c_). In this simulation work, the length and width of the solvent layer were equal to those of the crystal face layer, and the height of the solvent layer was determined by the number of molecules and the density of molecules contained in the solvent layer. A vacuum layer of 100 Å was added above the solvent layer to eliminate the effect of periodic boundary conditions in the *z*-axis direction. The sizes of the simulation boxes constructed for each crystal face system are shown in Tables S1 and S2.

#### 3.2.3. Molecular Dynamics Simulation Details

The simulation work in this paper was completed by using Materials Studio 2018 software. The molecular dynamics simulation of the crystal surface–solvent model system was carried out. The NVT ensemble was used, and the initial particle velocity was randomly assigned at 298 K. The simulation time was 500 ps with a time step of 1 fs, 1 frame of simulation trajectory was output every 100 steps, and a total of 5000 frames of simulation trajectory was obtained. The Andersen temperature control method was used to control the temperature of the system. Under the COMPASS force field, the atomic charge calculation method is forcefield assigned. The electrostatic interactions were calculated using the Ewald summation method, and the van der Waals interactions were calculated using an atom-based method with a truncation distance of 12.5 Å.

## 4. Conclusions

The crystal morphology of ISMN under vacuum was predicted using the AE model, and molecular dynamics simulations were performed for the model system containing morphologically important growth surfaces. The crystal morphology was predicted in two solvent systems, CH_2_Cl_2_ and CH_2_Cl_2_-C_6_H_14_, and the reasons for the changes in crystal morphology were analyzed. The conclusions are summarized as follows:

1. In the CH_2_Cl_2_ solvent system, the type of interaction present at each crystal face was determined using RDF analysis. Hydrogen bond interactions determine the crystal morphology to a certain extent, with the bond length, bond angle, and number of hydrogen bonds affecting the crystal morphology.
2. The use of C_6_H_14_ as a volatile agent accelerates crystal growth and increases the diffusion rate of CH_2_Cl_2_ molecules; the addition of C_6_H_14_ affects the interaction between the crystal face layer and the solution layer mainly by influencing the number of hydrogen bonds in the vicinity of the crystal face.
3. The addition of C_6_H_14_ did not have a major effect on the overall morphology of the crystals, mainly because the three morphologically important growth faces, (1 0 0), (1 1 0), and (1 1 1), are still dominated by CH_2_Cl_2_ solvent molecules in the vicinity of the crystal faces.
4. In the CH_2_Cl_2_-C_6_H_14_ solvent system, the difference in density distribution near the (1 0 −1) crystal face may account for the reappearance of the (1 1 −1) crystal face as a morphologically important growth surface.

## Figures and Tables

**Table 1:** Parameters associated with morphologically important growth surfaces in the ISMN.

(h k l)	Multiplicity	d_hkl_ (Å)	E_att_ (kcal/mol/Unit Cell)	Total Habit Facet Area (Å^2^)	Total Habit Facet Area Percentage
(1 0 0)	4	15.42	−48.81	26,086.60	72.56%
(1 1 0)	4	10.90	−61.87	4621.26	12.85%
(1 0 1)	4	6.05	−177.07	1339.40	3.73%
(1 0 −1)	4	6.05	−177.07	1339.40	3.73%
(1 1 1)	4	5.63	−170.28	1282.82	3.57%
(1 1 −1)	4	5.63	−170.28	1282.82	3.57%

**Table 2:** Calculation results of crystal face rugosity S.

(h k l)	A_hkl_ (Å^2^)	A_acc_ (Å^2^)	S
(1 0 0)	101.342	131.615	1.299
(1 1 0)	143.319	202.505	1.413
(1 1 1)	277.607	392.680	1.415
(1 1 −1)	277.607	391.540	1.410
(1 0 1)	258.448	340.865	1.319
(1 0 −1)	258.448	356.375	1.379

**Table 3:** Calculation results of E′_att_ in the CH_2_Cl_2_ solvent system.

(h k l)	d_hkl_ (Å)	E_att_ (kcal/mol/Unit Cell)	Z_cry_	Z_hkl_	A_hkl_ (Å^2^)	A_box_ (Å^2^)	E_int_ (kcal/mol)	E’_att_ (kcal/mol/Unit Cell)
(1 0 0)	15.42	−48.81	8	8	101.34	2432.21	−577.44	−24.75
(1 1 0)	10.90	−61.87	8	8	143.32	3439.66	−919.91	−23.54
(1 1 1)	5.63	−170.28	8	8	277.61	6663.03	−2211.43	−78.15
(1 1 −1)	5.63	−170.28	8	8	277.61	6663.03	−2074.29	−83.86
(1 0 1)	6.05	−177.07	8	8	258.45	2326.03	−805.57	−87.56
(1 0 −1)	6.05	−177.07	8	8	258.45	2326.03	−840.60	−83.67

**Table 4:** Hydrogen bond statistics in the CH_2_Cl_2_ solvent system.

(h k l)	HB Length ^a^ (Å)	HB Angle ^b^ (°)	S_1_% ^c^ (HB Length < 2.5 Å)	S_2_% ^d^ (HB Angle > 150°)	N_HB_ ^e^	N_H1_ ^f^	N_HB-per H1_ ^g^	A_box_ ^h^(Å^2^)	N_HB-unit area_ ^i^	A_hkl_ ^j^ (Å^2^)	N_HB-hkl_ ^k^
(1 0 0)	2.52	147.13	50.00%	44.67%	24.80	18	1.38	2432.21	0.0102	101.342	1.03
(1 1 0)	2.57	144.13	42.34%	31.99%	47.19	48	0.98	3439.66	0.0137	143.319	1.97
(1 1 1)	2.59	147.53	37.49%	45.55%	20.92	24	0.87	6663.03	0.0031	277.607	0.87
(1 1 −1)	2.56	144.21	43.60%	31.93%	49.50	48	1.03	6663.03	0.0074	277.607	2.06
(1 0 −1)	2.56	144.28	43.80%	30.99%	17.68	18	0.98	2326.03	0.0076	258.448	1.96

^a^ HB length is the average hydrogen bond length in Å. ^b^ HB angle is the average hydrogen bond angle in °. ^c^ S_1_% is the percentage of hydrogen bond lengths less than 2.5 Å. ^d^ S_2_% is the percentage of hydrogen bonding angles greater than 150°. ^e^ N_HB_ is the average number of hydrogen bonds contained in each frame of the trajectory. ^f^ N_H1_ is the number of H1 atoms involved in the formation of hydrogen bonds in the simulated trajectory. ^g^ N_HB-per H1_ is the average number of hydrogen bonds formed per H1 atom. ^h^ A_box_ is the simulated box cross-sectional area in Å^2^. ^i^ The N_HB-unit area_ is the average number of hydrogen bonds formed per unit crystal face area. ^j^ A_hkl_ is the cross-sectional area of the crystal face in the unit cell. ^k^ N_HB-hkl_ is the average number of hydrogen bonds contained in each crystal face in a unit cell.

**Table 5:** Results of MAE calculations in the CH_2_Cl_2_-C_6_H_14_ solvent system.

(h k l)	d_hkl_ (Å)	E_att_ (kcal/mol/Unit Cell)	Z_cry_	Z_hkl_	A_hkl_ (Å^2^)	A_box_ (Å^2^)	E_int_ (kcal/mol)	E’_att_ (kcal/mol/Unit Cell)
(1 0 0)	15.42	−48.81	8	8	101.34	2432.21	−556.13	−25.63
(1 1 0)	10.90	−61.87	8	8	143.32	3439.66	−903.67	−24.22
(1 1 1)	5.63	−170.28	8	8	277.61	6663.03	−2126.07	−81.70
(1 1 −1)	5.63	−170.28	8	8	277.61	6663.03	−1991.14	−87.33
(1 0 1)	6.05	−177.07	8	8	258.45	2326.03	−766.75	−91.87
(1 0 −1)	6.05	−177.07	8	8	258.45	2326.03	−786.70	−89.66

**Table 6:** Diffusion coefficients of CH_2_Cl_2_ in two solvent systems.

(h k l)	D_α1_^a^ (10^−8^ m^2^/s)	D_α2_^b^ (10^−8^ m^2^/s)
(1 0 0)	0.2661	0.3192
(1 1 0)	0.2650	0.3657
(1 1 1)	0.3526	0.4465
(1 1 −1)	0.3773	0.4369
(1 0 1)	0.2670	0.3475
(1 0 −1)	0.3038	0.3673

^a^ D_α1_ is the diffusion coefficient of CH_2_Cl_2_ in the CH_2_Cl_2_ solvent systems. ^b^ D_α2_ is the diffusion coefficient of CH_2_Cl_2_ in the CH_2_Cl_2_-C_6_H_14_ solvent systems.

**Table 7:** Hydrogen bonding statistics in the CH_2_Cl_2_-C_6_H_14_ solvent system.

(h k l)	HB Length ^a^ (Å)	HB Angle ^b^ (°)	S_1_% ^c^ (HB Length < 2.5 Å)	S_2_% ^d^ (HB Angle > 150°)	N_HB_ ^e^	N_H1_ ^f^	N_HB-per H1_ ^g^	A_box_ ^h^ (Å^2^)	N_HB-unit area_ ^i^	A_hkl_ ^j^ (Å^2^)	N_HB-hkl_ ^k^
(1 0 0)	2.51	147.27	50.49%	45.16%	22.87	18	1.27	2432.21	0.0094	101.342	0.95
(1 1 0)	2.55	144.49	44.53%	33.08%	40.29	48	0.84	3439.66	0.0117	143.319	1.68
(1 1 1)	2.59	147.76	37.75%	45.95%	20.72	24	0.86	6663.03	0.0031	277.607	0.86
(1 1 −1)	2.57	143.93	43.01%	31.22%	39.87	48	0.83	6663.03	0.0060	277.607	1.66
(1 0 −1)	2.55	144.61	45.73%	31.60%	15.85	18	0.88	2326.03	0.0068	258.448	1.76

Note: The meanings of the physical quantities marked by superscript letters (a–k) in the table are the same as in Table 4.

**Table 8:** Comparison of cell lattice parameters.

Lattice Parameter	a (Å)	b (Å)	c (Å)	α (°)	β (°)	γ (°)
Primitive unit cell	15.992	15.992	6.523	90	90	90
Cambridge Structural Database	15.926	15.926	6.509	90	90	90
COMPASS	15.419	15.419	6.572	90	90	90
Relative error	3.58%	3.58%	0.75%	0.00%	0.00%	0.00%

## Data Availability

Data are contained within the article or Supplementary Materials.

## Supplementary Materials

The following supporting information can be downloaded at: www.mdpi.com/xxx/s1, Table S1: The size of the simulated box and the number of solvent molecules contained in the CH_2_Cl_2_ solvent system; Table S2: The size of the simulated box and the number of solvent molecules contained in the CH_2_Cl_2_-C_6_H_14_ solvent system.
