# Supplementary material for: The Effect of Solvents on the Crystal Morphology of Isosorbide Mononitrate and Its Molecular Mechanisms
Source: Molecules. 2024 Jan 11;29(2):0. doi: 10.3390/molecules29020367 (PMC11154549; doi:10.3390/molecules29020367)
Supplement: Supplementary file 1 [file molecules-29-00367-s001.zip › molecules-2766688-supplementary.pdf]

Supplementary Material for: The Effect of Solvents on the Crystal Morphology of Isosorbide Mononitrate and its Molecular Mechanisms

The size of the simulation box constructed for each crystal face system and the number of molecules contained are shown below.

Table S1 The size of the simulated box and the number of solvent molecules contained in the CH_2Cl_2 solvent system

(h k l)	a (Å)	b (Å)	c (Å)	T_c ^{a}(Å)	T_s ^{b}(Å)	α (°)	β (°)	γ (°)	N_{CH2Cl2}
(1 0 0)	46.26	52.58	189.27	30.84	43.75	90	90	90	1000
(1 1 0)	52.58	65.42	179.98	32.71	30.93	90	90	90	1000
(1 1 1)	99.38	67.05	180.20	22.52	39.92	90	90	90	2500
(1 1 -1)	99.38	67.05	180.46	22.52	39.92	90	90	90	2500
(1 0 1)	50.28	46.26	185.06	24.18	45.75	90	90	90	1000
(1 0 -1)	46.26	50.28	187.79	24.18	45.74	90	90	90	1000

[a] T_c is the thickness of the crystal face layer in Å.

[b] T_s is the thickness of the solvent layer in Å.

Table S2 The size of the simulated box and the number of solvent molecules contained in the CH_2Cl_2-C_6H_{14} solvent system

(h k l)	a (Å)	b (Å)	c (Å)	T_c ^{a}(Å)	T_s ^{b}(Å)	α (°)	β (°)	γ (°)	N_{CH2Cl2}	N_{C6H14}
(1 0 0)	46.26	52.58	201.54	30.84	53.60	90	90	90	817	200
(1 1 0)	52.58	65.42	190.26	32.71	37.90	90	90	90	817	200
(1 1 1)	99.38	67.05	190.89	22.52	48.89	90	90	90	2041	500
(1 1 -1)	99.38	67.05	191.17	22.52	48.89	90	90	90	2041	500
(1 0 1)	50.28	46.26	196.53	24.18	56.05	90	90	90	817	200
(1 0 -1)	46.26	50.28	200.68	24.18	56.05	90	90	90	817	200

Note: The meanings of the physical quantities in the table are the same as in **Table S1**.
